# Dynamic Magnetic Resonance Imaging Demonstrates the Integrity of Perineal Reconstruction following Cylindrical Abdominoperineal Excision with Reconstruction of the Pelvic Floor Using Porcine Collagen

**DOI:** 10.1155/2012/752357

**Published:** 2012-01-22

**Authors:** D. O. Kavanagh, H. Imran, A. Almoudaris, P. Ziprin, O. Faiz

**Affiliations:** ^1^Department of Surgery, Mayo General Hospital, Castlebar, Mayo, Ireland; ^2^Department of Surgery, St Mary's Hospital, Praed Street, London W2 1NY, UK; ^3^Academic Surgical Unit, St Mary's Hospital, Praed Street, London W2 1NY, UK

## Abstract

A 72-year-old female presented with a six-month history of increased frequency of defecation, rectal bleeding, and severe rectal pain. Digital rectal examination and endoscopy revealed a low rectal lesion lying anteriorly. This was confirmed histologically as adenocarcinoma. Radiological staging was consistent with a T_3_N_2_ rectal tumour. Following long-course chemoradiotherapy repeat staging did not identify any metastatic disease. She underwent a laparoscopic cylindrical abdominoperineal excision with *en bloc* resection of the coccyx and posterior wall of the vagina with a negative circumferential resection margin. The perineal defect was reconstructed with Permacol (biological implant, Covidien) mesh. She had no clinical evidence of a perineal hernia at serial followup. Dynamic MRI images of the pelvic floor obtained during valsalva at 10 months revealed an intact pelvic floor. A control case that had undergone a conventional abdominoperineal excision with primary perineal closure without clinical evidence of herniation was also imaged. This confirmed subclinical perineal herniation with significant downward migration of the bowel and bladder below the pubococcygeal line. We eagerly await further evidence supporting a role for dynamic MR imaging in assessing the integrity of a reconstructed pelvic floor following cylindrical abdominoperineal excision.

## 1. Introduction

National and international data on abdominoperineal excision (APER) reveal high-margin positivity rates and a high incidence of intraoperative perforation [1–3]. Endeavours to improve outcomes in rectal cancer surgery have focussed upon the use of total mesorectal excision and preoperative chemoradiotherapy [4, 5]. Despite these efforts, outcomes following APER have been consistently poor. Using tissue morphometry, Marr and colleagues have described the tapering or “coning” of the specimen at the level of the pelvic floor that occurs with the conventional total mesorectal excision technique and can yield a positive circumferential resection margin (CRM) [6]. Holm and coworkers reported their initial experience in 2007 with the “cylindrical” or “extralevator” excision technique [7]. This results in the elimination of Morson's waist and converts a coned specimen into a cylindrical specimen with consistent data demonstrating a reduction in intraoperative perforation and CRM positivity rates [8]. However, this also results in a significant perineal defect in previously irradiated tissue. Intuitively this will result in higher perineal morbidity rates and higher perineal herniation rates. Preliminary data from the extralevator group highlights this challenge and suggests that reconstruction of the pelvic floor may avoid this [8]. Techniques advocated for closing the perineal defect include a gluteal flap, vertical rectus abdominis myocutaneous (VRAM) flap, or other tissue reconstruction. Plastic surgical procedures however require additional operative time and the availability of a plastic surgeon. The availability of biological mesh implants has led to an alternative method in pelvic floor reconstruction. The long-term durability of this in the setting of perineal reconstruction remains to be elucidated. Clearly, clinical assessment will identify obvious herniation but is subjective. In order to establish it as the standard approach to perineal reconstruction after APER and justify the cost of the implant, we feel it is appropriate to objectively measure the integrity of the pelvic floor during the postoperative followup.

## 2. Case Report

A 72-year-old female was referred with a 6-month history of faecal urgency, rectal bleeding, and severe rectal pain. Colonoscopy revealed an anterior rectal mass. The lower border of the rectal lesion was palpable at 3 centimeters from the anal verge and encroached on the rectovaginal septum. Histopathological examination of biopsy specimens confirmed rectal adenocarcinoma. Computerised tomography of the abdomen and pelvis did not reveal distant metastases. MRI pelvis staging reported a T_3_N_2_ tumor with enlarged inguinal nodes. However, fine needle aspiration of the latter was negative. Following discussion at the multidisciplinary meeting the patient was referred and underwent a six-week course of chemoradiotherapy. Following a subsequent 6-week interval the patient was admitted electively for laparoscopic abdominoperineal excision (with cylindrical perineal excision). The abdominal component was performed supine. Ports were placed to facilitate a medial-to-lateral approach to the left colon. The inferior mesenteric artery was isolated and divided using an endovascular stapler (Ethicon) with prior identification of the left ureter. The medial dissection was completed separating the mesocolic fascia from Gerota's fascia. The lateral peritoneal attachments were divided in a cephalad direction with adequate mobilisation to facilitate adequate length to form an end sigmoid colostomy. This ladies' body habitus required proximal mobilisation to facilitate colostomy formation. The pelvic dissection was commenced along the plane of the mesorectal envelope. The anterior landmark for cessation of abdominal dissection was the point at which the vagina was initially encountered. The posterior dissection ended at the corresponding posterior level at the upper border of the coccyx to ensure that the mesorectum was not fully mobilised off the pelvic, thereby avoiding “coning”. The sigmoid colon was divided laparoscopically, and an end colostomy was constructed in the left iliac fossa.

The perineal component was undertaken in the prone “jack-knife” position. The anal orifice was closed using a purse-string suture. The anus and sphincter complex was widely excised with cephalad extension of the incision to facilitate access to the coccyx. The sacrococcygeal joint was disarticulated, and the pelvic peritoneum was breached posteriorly. Lateral dissection across the ischiorectal fossae was then performed with wide division of the levators to meet the abdominal dissection. Finally the specimen was delivered, and the anterior component was completed with *en bloc* resection of the posterior vaginal wall as the tumor had initially encroached on the rectovaginal septum.

The vaginal wall was closed, and the pelvic floor defect was reconstructed using a Permacol Biologic Implant (porcine dermal collagen) which was fixed to the pelvic floor defect circumferentially using interrupted 2/0 polypropylene sutures. Initial suture placement was laterally along the fascial line of attachment of the pelvic floor muscle. Posteriorly it was sutured to the fascia posterior to the sacrococcygeal junction. Anteriorly it was sutured either side of the midline so that it did not cause further distortion of the posterior vaginal wall. The perineal wound was closed in three layers with interrupted absorbable sutures. A closed suction drain was left in situ. The patient made a favourable postoperative recovery and was discharged home on the 13th postoperative day. She lived alone, and at the time of discharge she was fully independent in her stoma care. Her perineal wound was fully healed. Final histology revealed residual poorly differentiated adenocarcinoma with associated cytological changes characteristic of neoadjuvant therapy. Extensive fibrosis was noted indicative of favourable regression in response to neoadjuvant therapy. Extramural venous invasion was observed. All 14 lymph nodes retrieved showed no evidence of malignancy, and the circumferential resection margin was 3 mms with no evidence of invasion into the vaginal wall.

**Figure 1:**
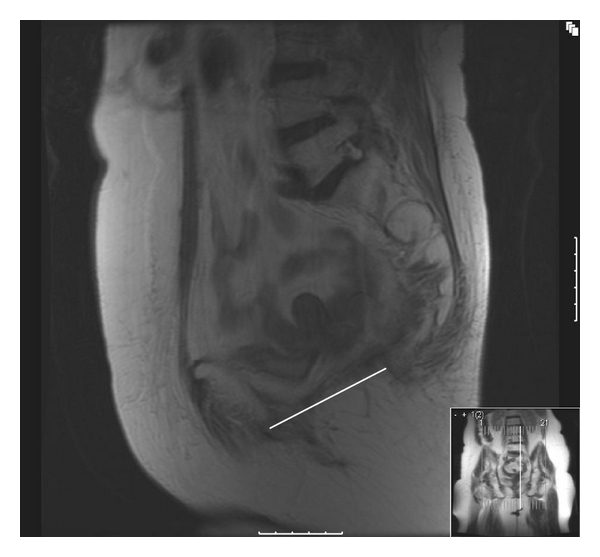
T_2_-weighted saggital view. Patient lying supine with breath held. White line represents “modified pubococcygeal” line. The pelvic floor has been replaced by Permacol following cylindrical abdominoperineal excision.

**Figure 2:**
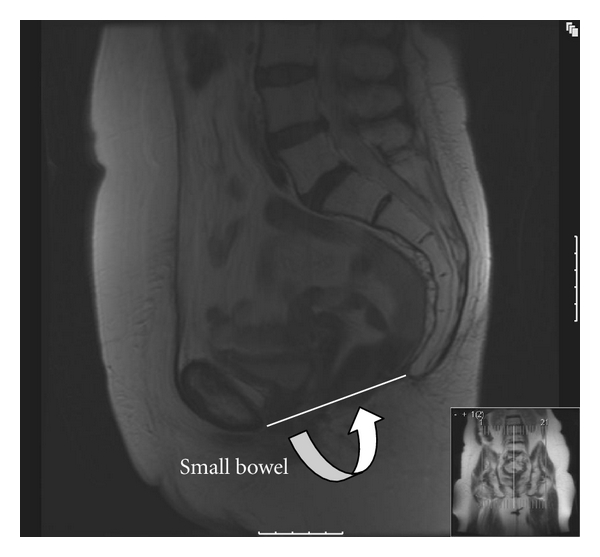
T_2_-weighted saggital view. Patient in supine position performing a Valsalva. The modified pubococcygeal line is seen. The downward migration of the small bowel (curved arrow) below this line is <1 cm indicating an intact reconstructed pelvic floor.

**Figure 3:**
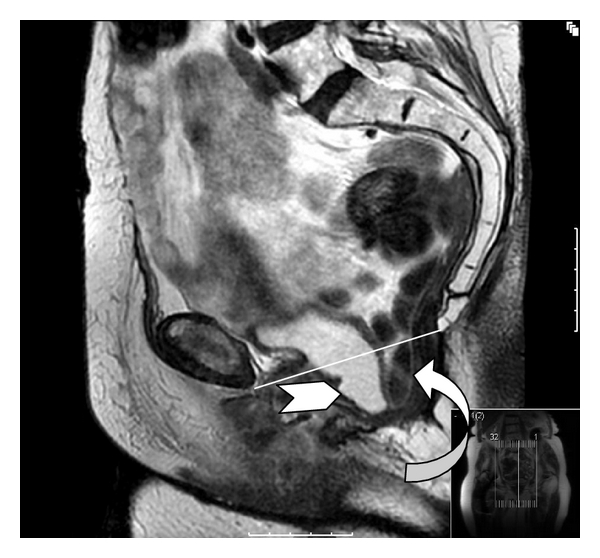
T_2_-weighted saggital view. Patient underwent a conventional abdominoperineal excision with primary closure of the pelvic floor. Images are obtained in a supine position with the patient performing a Valsalva. There is abnormal descent (>1 cm) of the pelvic floor with posteroinferior herniation of the bladder (arrowhead) and small bowel (curved arrow) below the pubococcygeal line.

A dynamic MRI was performed at 10 months following surgery. This was performed initially at rest with breath hold (no valsalva), and 5-millimeter T_2_-weighted sagittal and coronal views were obtained (Figure 1). It was repeated with valsalva (breath hold and pushing down), and the same images were acquired (Figure 2). This demonstrates the Permacol implant *in situ *along the “modified” pubococcygeal line. This anatomical plane runs from the inferior border of the pubis symphysis to the lower border of S_5_ and is a variant to facilitate description of pelvic floor integrity following coccygeal excision. It represents the normal level of the pelvic floor which in this case has been reconstructed. In the setting of an intact pelvic floor the most inferior aspect of the descending intra-abdominal contents should lie <1 cm below the modified pubococcygeal line as illustrated in Figure 2. A control image was obtained from a straining patient who underwent a conventional abdominoperineal excision without pelvic floor reconstruction who had no clinical evidence of a perineal hernia (Figure 3). This reveals significant inferior migration of the pelvic floor with the bladder and small bowel lying >1 cm below the pubococcygeal line. The patient remains well at 12-month followup with an intact perineal wound.

## 3. Discussion

This paper illustrates the feasibility of using dynamic MRI scanning to image perineal integrity following extralevator abdominoperineal excision and subsequent porcine mesh reconstruction. Ideally, the abdominoperineal resection technique must serve the oncological requirement to satisfactorily clear malignant disease with minimal morbidity. Much of the morbidity that commonly follows this procedure arises from the perineum which has typically undergone radiotherapy. Intuitively the larger defect created will yield higher perineal morbidity, and initial experiences support this hypothesis [7, 8]. Options to reconstruct include flaps and biological meshes. The former requires additional operative time and the availability of a plastic surgeon. Christensen et al. recommend biological mesh over flap reconstruction as this yields higher healing rates and lower hernia [9]. This is based on clinical followup. In other clinical settings the long-term durability of biological meshes has been challenged [10]. We feel it would be optimal to justify the widespread application of biological meshes in this setting by objective measurement of the pelvic floor integrity using dynamic MR imaging as they are costly. This is the approach we have adopted in the current case.

Sir Ernest Miles described many operative procedures but he had a particular interest in the area of rectal cancer [11]. Early in the 20th century, most patients with rectal cancer underwent perineal procedures to address advanced, symptomatic disease. Miles believed that rectal cancer spread laterally and downwards and emphasised the need for a wide excision when performing an abdominoperineal excision. His description of the procedure comprised undertaking the abdominal component, fashioning a colostomy and then closing the pelvic peritoneum. Interestingly, he also described performing a wide perineal dissection across the levator ani to retrieve the specimen. Operative mortality was high, and he reported a local recurrence rate of 28% in his series [11]. The evolution of rectal cancer management, including an endeavour to reduce local recurrence rates, has led to various modifications of Miles' original approach. Improvements in operative haemostasis and antisepsis have facilitated a reduction in operative mortality. Total mesorectal excision, popularised by Heald and colleagues [2]. improved outcome for rectal cancers treated by anterior resection but local recurrence rates for abdominoperineal excision remained unacceptably high despite radiotherapy prior to surgery [8]. The concept of the circumferential resection margin involvement led Marr and coworkers to analyse specimens from the Dutch TME trial [6]. They observed that specimens tended to “cone” at the level of the levators (the typical tumor level in a patient requiring an APER) when a TME was performed, and hence these patients had a positive circumferential resection margin (CRM) with predictably poorer outcomes [6]. Holm and colleagues introduced the concept of excising a cylinder of tissue by stopping the abdominal dissection at the level of the ischial spines before the mesorectum tapers in at the level of the pelvic floor and then completing the operation via the perineal approach [7]. This approach has been shown by the extralevator study group to reduce the incidence of CRM positivity [8]. We await with interest long-term survival and recurrence data on the clinical benefit of this improved pathological outcome.

One potential drawback to cylindrical excision is that the pelvic defect that follows the procedure could be associated with greater perineal morbidity than the conventional operation if not coupled with suitable perineal reconstruction. Several authors have utilised muscle flaps to close the perineal defect following cylindrical excision with favourable outcomes in individual personal series. Some surgeons report using vertical rectus abdominis myocutaneous (VRAM) and inferior gluteal artery perforator flaps (iGAP) [12–15]. Holm and colleagues in their landmark paper describing the concept of a cylindrical specimen reported perineal morbidity in 4/28 patients following reconstruction with a gluteal flap [7]. These techniques can be time consuming and require the availability of a plastic surgeon for optimal outcomes. This can add considerable cost and operative time particularly if an abdominal wall flap is used in the prone patient.

The advent of biological meshes provides a novel approach to reconstructing the pelvic floor. The xenograph composed of acellular porcine dermis has yielded positive postoperative outcomes across multiple surgical disciplines during its preclinical trials and subsequent decade of clinical applications. Clinical data has revealed its safety profile in direct contact with small intestine while clinically evident infections can be safely treated with antimicrobial therapy and typically do not require removal in the setting of infection [16–21]. These benefits including a reduction in operative time must be offset against its cost. Early experience with biological implants to reconstruct following APE report favourable wound infection rates but prolonged perineal discomfort in over 50% of patients which eventually resolved at 8 months of followup (Table 1). Christensen et al. report favourable short-term outcomes with biological meshes compared to VRAM flap in a nonselected comparative series. This clinically assessed for perineal herniation in all cases.

Conventional APE involves a sutured closure of the pelvic defect with subsequent closure of the overlying fat and perineal skin. The classical procedure is however associated with significant perineal wound-related problems [22–25]. Specifically, case series suggest that perineal complications occur between 16 and 35% of cases. Figure 3 illustrates the mechanical perineal deficit visible in a patient that underwent a conventional APE (i.e., noncylindrical). The dynamic MRI images demonstrate significant downward descent and prolapse of the pelvic contents occurring upon straining. This may not always be clinically evident. Short-term perineal morbidity rates of up to 38% have been recorded after extralevator APE [10]. Improving oncological outcomes in this group of patients is however paramount. Hence this radical approach is likely to stay. Intuitively, the larger defect created with the extralevator approach is likely to lead to greater perineal morbidity if closed primarily. Reconstruction with either tissue flaps or biological mesh may however mitigate morbidity risk. Shaikh et al. demonstrated a nonsignificant reduction in short-term perineal wound morbidity when the pelvic floor was reconstructed with a muscle flap, 33% *versus* 43%: *P* = 0.24 [10]. Identification of superiority of either technique will necessitate a clinical trial. Dynamic imaging of the pelvic floor following reconstruction, as illustrated in this case, can demonstrate the integrity of the repaired defect. Correlation of radiological and clinical outcome may inform on the causation of perineal morbidity.

The current case highlights some of the challenges faced with attempts to improve outcome in low rectal cancer surgery. The large perineal defect that follows extralevator APE is likely to benefit from reconstruction. The clinical superiority of a single reconstructive technique that leads to reduced perineal morbidity has not been established. The functional integrity of the reconstructed pelvic floor can be clearly imaged using dynamic magnetic resonance imaging. This radiological approach could support an evaluation of outcome following differing types of perineal reconstruction in a larger series.

## Figures and Tables

**Table 1:** Data describing reconstruction with a biological implant following cylindrical abdominoperineal excision.

Author	Year	*n*	Biological implant	Short-term outcomes
Christensen et al. [9]	2010	11	Permacol	Not described
				Wound infection (1)
Sinna et al. [15]	2010	12	HADM^†^	Seroma (1)
				Chronic perineal pain (4)*
Wagstaff et al. [14]	2009	1	Surgisis	Not described
Boereboom et al. [16]	2009	11	Permacol	Infection and removal (1)
				Chronic pain (6)*
Han et al. [17]	2008	7	Permacol	Infection (1)

^†^Human acellular dermal matrix.

*Complete resolution at 6-month followup.
